# The relationship between novelty of introducing breaking at the 2024 Paris Olympics and potential viewers’ media engagement, anticipation, and viewing intention: a study

**DOI:** 10.1186/s40359-025-02655-7

**Published:** 2025-04-23

**Authors:** Seung-jae Lim, Sun-young Lim, Jeoung-hak Lee

**Affiliations:** 1 Department of Sports Marketing, Kyung Hee University, Yongin, 211732 Republic of Korea; 2 Department of Sports Marketing, Kyung-Hee University, Yongin, 211732 Republic of Korea

**Keywords:** 2024 Paris Olympics, Breaking, Young generation, Novelty, Anticipation, Media engagement, Viewing intention

## Abstract

**Background:**

This empirical study investigates the impact of various variables on the potential novelty and adoption level of breaking as an official event in the 2024 Paris Olympics and how these factors influence media engagement and viewing intentions. This is meaningful in providing academic implications that can predict potential consumer sports-viewing patterns post-COVID-19 by delving into the psychological shifts among consumers.

**Methods:**

This research involved 520 South Korean youths, potential viewers of the 2024 Paris Olympics, as participants. The final analysis incorporates responses from 493 participants after excluding 27 inadequate questionnaires. Analytical techniques such as frequency analysis, confirmatory factor analysis, reliability analysis, correlation analysis, and structural equation model analysis using SPSS 28.0 and AMOS 25.0 are employed.

**Results:**

The findings highlight several key points. First, the novelty of the introduction of breaking as an Olympic sport has a statistically significant effect on the anticipation, media engagement, and viewing intention of potential viewers. Second, while the anticipation of the introduction of breaking does not have a statistically significant effect on the viewing intentions of potential viewers, it does have a statistically significant impact on their media engagement. Finally, media engagement had a statistically significant effect on their viewing intentions.

**Conclusion:**

Breaking, which was introduced as a new sport at the 2024 Paris Olympics, was perceived by the younger generation as new, novel, and fresh, unlike the traditional Olympic image. This is consistent with the reason why the IOC adopted breaking as an Olympic sport. The results suggest that the novelty of introducing a new sport as an Olympic event has a positive effect on younger generations' perception of the Olympics and their potential viewing intentions.

**Supplementary Information:**

The online version contains supplementary material available at 10.1186/s40359-025-02655-7.

## Introduction

The modern era is changing sports and viewers’ attitude toward them. While baseball, which was traditionally considered a sport, was excluded from the Olympics, and weightlifting was on the verge of being kicked out, breakdancing or breaking was adopted as the official event at the 2024 Paris Olympics.

Breaking refers to a free-form dance in New York's street culture, centered on people of color who immigrated to the US in the 1970s [1]. Currently, breaking is called “B-boying, a genre of intangible art that expresses beauty using the body and has been established as a popular culture representing youth worldwide.” Breaking was first introduced as a demonstration sport at the 2018 Buenos Aires Youth Olympics, drawing in 30,000 young people. In response, IOC (International Olympic Committee) President Thomas Bach said, “The new sports proposed by the Paris Olympic Organizing Committee align with the 2020 Olympic agenda and provide an opportunity to communicate with the younger generation.” This change reflects the IOC's initiative to encourage young generations who dislike traditional sports to watch the Olympics [2].

**Fig. 1:**
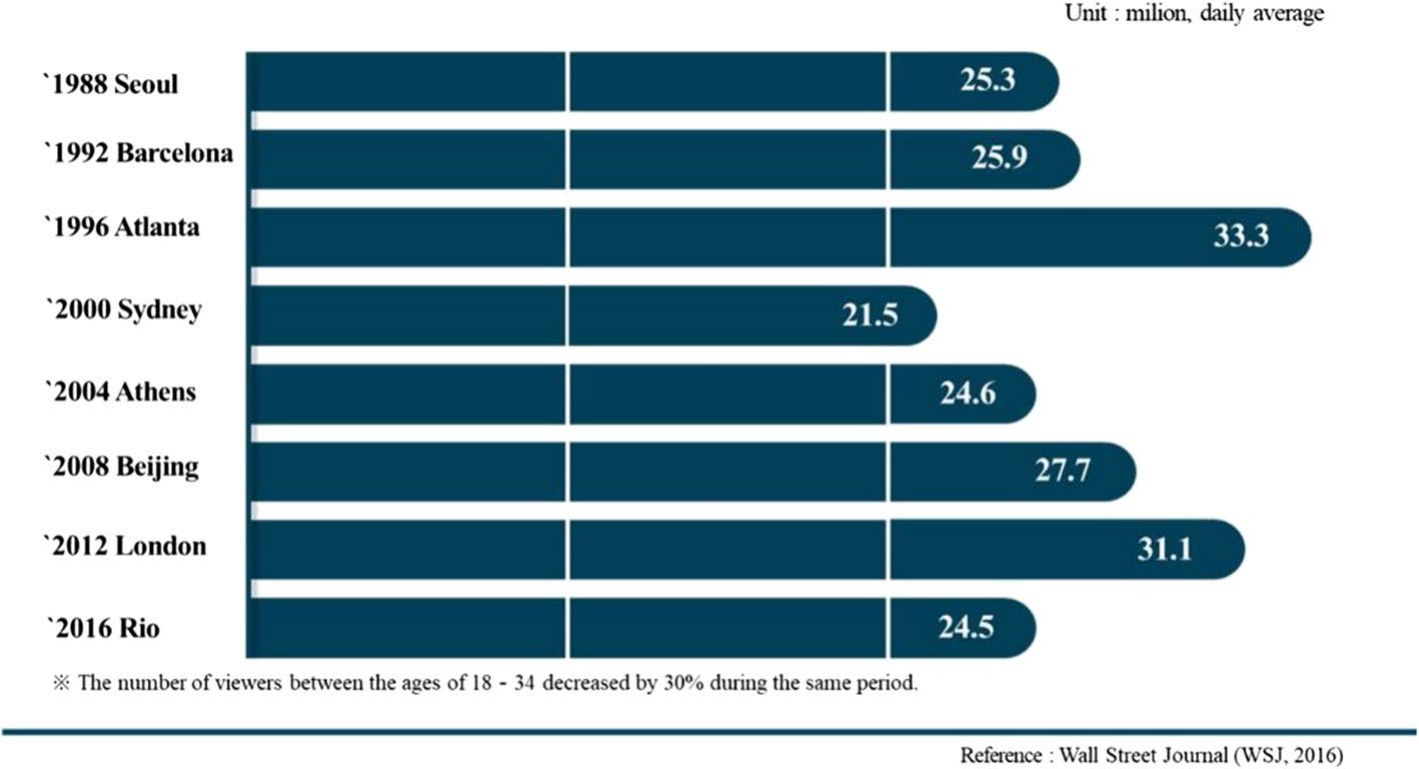
The number of viewers Olympic broadcasts in US (Source: Wall Street Journal, 2016)

The IOC's decision to break the traditional “grammar of sports” and adopt a number of sports popular among the younger generation as new Olympic sports is interpreted as a long-term strategic approach to secure the current younger generation as the main viewers [3]. Compared with the 2012 London Olympics, a survey by the *Wall Street Journal* found that the number of viewers aged 18–34 decreased by 30% during the same period as the 2016 Rio Olympics, which showed an 18% decline in viewership ratings, demonstrating that the Olympics could no longer hold the interest and consensus of younger generations (Fig. 1). Moreover, the IOC relies on broadcasting rights for 73% of its total income; thus, as the main consumer, the younger generation turning away from broadcasting increases the possibility of the source of income faltering [4–6]. Finally, the recent introduction of breaking by the IOC as a new event favored by the young generation is interpreted as a long-term strategic approach to securing the current young generation of consumers [7].

Recent consumption trends of the younger generation include new experiences, fun, and value-driven pursuits [8]. In recent years, the changing consumption trends of the younger generation have been instrumental in the adoption of skateboarding, sports climbing, surfing, 3 × 3 basketball, and BMX freestyle as official Olympic events at the 2020 Tokyo Olympics [9]. The current young generation, referred to as the Millennials and Gen Z (millennials + generation Z), has begun to take an interest in adventurous sports that go beyond the stage of simply participating in sports activities and finding satisfaction and can develop into extreme achievement and self-realization through overcoming more creative and extreme situations [10]. This influence has led to a rise in the popularity of extreme sports, and many sports previously considered youth subcultures are now featured in the world's biggest sporting event, the Olympics. In this context, the novelty of breaking being adopted as an official Olympic sport symbolizes strengthening the potential viewing intention of the Millennials and Gen Z who feel bored with traditional sports. For example, at the 2019 World Martial Arts Masterships held in Chungju, South Korea, there were events both familiar (e.g., Taekwondo) and somewhat unfamiliar to tourists [11]. Accordingly, the organizers held a program where tourists could experience unfamiliar martial arts such as wrestling, martial arts practice, pencak silat, kabaddi, kurashi, sambo, and savate, thereby, increasing understanding of martial arts from around the world while encouraging voluntary participation from tourists and providing them with unforgettable and novel experiences [12]. This is considered an exemplary case of appropriately utilizing the novelty of a sports event. According to the results report of the competition, 60.9% of all respondents, including the participating athletes and spectators, were satisfied with the overall aspects of the competition, confirming the possibility of it developing into a sustainable sports event [13]. Sports viewing behavior is triggered by emotional responses to excitement [14]. Therefore, the novelty of the Olympics acts as a powerful antecedent variable that raises anticipation for it by triggering a significant level of interest and response from potential viewers [15]. In this context, anticipation is defined as a strong belief in a successful future outcome [16, 17], and in this study, the concept of expectation was explained as a subjective perception that includes the public's hope for the future after breakdancing was adopted as an official Olympic event [18]. Based on these prior studies, the high level of anticipation formed by potential Olympic viewers is expected to enhance their media engagement and viewing intention in the process leading to strong satisfaction with future Olympics events [19].

However, despite the IOC’s efforts to attract the Millennials and Gen Z to the Olympics by introducing breaking, there is currently a severe lack of actual research and verification on whether this strategic attempt is being successful. Accordingly, approximately one year before the 2024 Paris Olympics, we judged that a study was necessary to empirically analyze the relationship between the perception of novelty of Olympic sports and potential viewing intention, reflecting the consumption trends of the Millennials and Gen Z, as a prerequisite for expanding the base of Olympic viewers. Therefore, this study attempts to provide practical implications by structurally analyzing the relationship between the novelty of breaking, which was introduced as an official event at the 2024 Paris Olympics, and the potential viewers' anticipation, media engagement, and viewing intention, reflecting the preferences of the Millennials and Gen Z.

## Theoretical background and hypotheses

### The relationship between novelty and anticipation

The relationship between novelty and anticipation novelty is a concept that contrasts with boredom felt by familiar and repetitive stimuli and refers to a unique experience [20, 21]. It arises from the desire and anticipation to experience something unprecedented when the level of stimulation in daily life falls short of the level sought by the individual, and boredom is felt [22–27]. Accordingly, Jeong and Kim [28] reported that the unique stimulation experienced by sports event tourists serves as a mechanism to increase the level of anticipation for sports viewing; Curran and Meuter [29] state that consumers who tend to explore and enjoy new environments and situations also show high levels of anticipation for consumption activities. Considering the results of the previous studies, the novelty of introducing breaking as an official Olympic event is expected to increase anticipations for the 2024 Paris Olympics.

- Hypothesis 1*The novelty of introducing breaking as an Olympic event has a significant effect on the anticipation of potential Olympic viewers.*

### The relationship between novelty and media engagement

It is, in general, acknowledged that the potential for continued development of sports exists in various media [14]. By utilizing the development technology of various media, the value of sports can be increased, and a competitive advantage can be secured [30]. In this context, media participation is defined as consumers’ active involvement and participation in media [31]. Previous studies on this topic have reported that the element of novelty has a positive effect on consumer engagement [32–35]. Specifically, Pack and Kim [32] presented results showing that the unique expressiveness of World Cup commentators provided fresh commentary to viewers, which had a positive effect on media participation, and Jeon [33] argued that the new experience about the North Korean cheering squad at the 2018 Pyeongchang Olympics stimulated the media participation of tourists. Moreover, Kim and Ha [34] and Oh [35] reported that new, unprecedented types of SNS information characteristics increase media engagement. Based on this, breaking is expected to be an important entertainment content that can induce strong media participation by creating a new perception among potential viewers watching the 2024 Paris Olympics that is different from existing Olympic events.

- Hypothesis 2*The novelty of introducing breaking as an Olympic event has a significant effect on the media engagement of potential Olympic viewers.*

### The relationship between novelty and viewing intention

Viewing intention refers to the planned future behavior of wanting to watch the Olympics where breaking is introduced as an official event [3] and is considered an indicator of the probability that it will turn into viewing behavior [36]. Chio et al. [3] found a relationship between novel and fresh elements of sports introduced in the 2020 Tokyo Olympics and potential viewing intentions, and Kayat et al. [37] found that the stronger the experience of fresh stimuli for a specific tourist destination, the higher the level of tourists' expectations, ultimately leading to positive consumption behavior patterns. In addition, Jeong and Kim [38] have presented results showing that novel experiences of spectators at spectator sporting events can increase their intention to revisit, and emphasized that novelty should be considered important in the decision-making process for predicting behavior. In the same context, Lim and Lim [39] state that as the desire for Olympic novelty increases because of the introduction of new sports, expectations for the Olympics also increase, which ultimately leads to viewing intention. Consequently, the novelty of the 2024 Paris Olympics, which will feature breakdancing, is expected to act as a catalyst to increase potential viewing intention.

- Hypothesis 3 *The novelty of introducing breaking as an Olympic event has a significant effect on the viewing intentions of potential Olympic viewers.*

### The relationship between anticipation and viewing intention

Tse and Wilton [40] state that anticipation is a judgment of the possibility of a specific event and a positive or negative evaluation of the event. In other words, consumer anticipation is the belief that consumers have about a specific service’s performance and serves as a standard or ground for evaluating actual service performance [41]. In this regard, Hsu, Chang, and Chen [42] stated that consumers' anticipation had a positive effect on their behavior, and Ahn [43] and Seo [44] also confirmed a significant relationship between anticipation of mega-sports events and sports participation behavior. Therefore, viewers' anticipation of breaking, which was adopted as an official event at the 2024 Paris Olympics, can be an important variable in predicting consumer behavior toward the target and will be closely related to the intention to watch the Olympics because of consumer behavior.

- Hypothesis 4 *The anticipation of introducing breaking as an Olympic event will have a significant effect on the viewing intentions of potential Olympic viewers.*

### The relationship between anticipation and media engagement

Having a high anticipation of a particular situation means that positive results can be brought about based on a strong belief in it [45]. Prior studies have revealed that high expectations lead to strong engagement [46], and media engagement is reported to act as a variable that results in the anticipation of sports events. Moreover, according to Jung [47], in the case of experience goods, it is difficult to judge quality before directly encountering the service; therefore, pre-established anticipation is important. Considering these points, the level of media engagement of potential viewers is assumed to be determined by their level of anticipation for break dancing, which was introduced as an official event at the 2024 Paris Olympics.

- Hypothesis 5
- * The anticipation of introducing breaking as an Olympic event will have a significant effect on the media engagement of potential Olympic viewers.*

### The relationship between media engagement and viewing intention

Viewing intention is an individual's willingness and belief in watching a sporting event on a media platform [48]. That is, the level of media engagement perceived by consumers in Olympics events can lead to their evaluation or viewing of the Olympics. Lim et al. [49] argued that social media engagement had a positive effect on sports channel viewing behavior in a study targeting viewers of the 2014 Sochi Winter Olympics in Korea. Kim et al. [50] confirmed that brand app engagement had a significant effect on brand app usage attitudes. In addition, Kim and Son [51] showed that media participation had a direct effect on attitudes toward use; accordingly, an increase in media participation by potential viewers of the 2024 Paris Olympics is expected to have a positive effect on consumption behavior.

- Hypothesis 6 *Media engagement has a significant effect on the viewing intentions of potential Olympic viewers.*

**Fig. 2:**
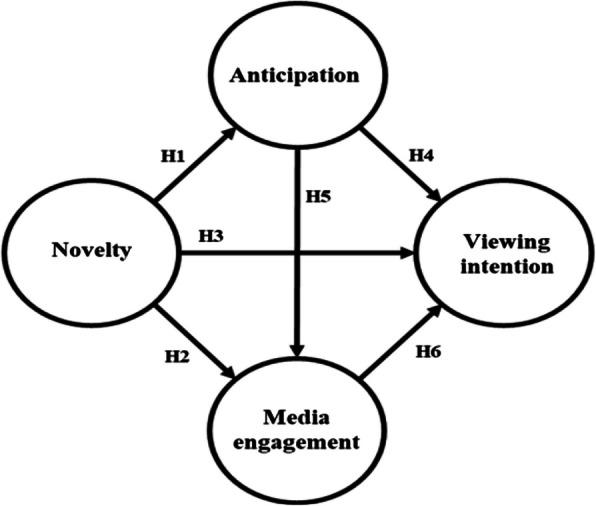
Research model

Based on the results of previous studies, the following hypotheses and models were established Fig. 2.

## Methods

### Participants

The survey was conducted from March to July 2022, and the subjects of this study were limited to younger generations between the ages of 17–30 years, residing in Seoul and Gyeonggi-do in 17 cities and provinces in South Korea. According to the Korea Professional Sports Association (KPSA), as of 2023, Seoul and Gyeonggi-do will have the highest percentage of spectators watching sports. Based on this, it was judged that a homogeneous sample of potential viewers of the 2024 Paris Olympics could be secured compared with other regions. In addition, the age limit is in line with the IOC's purpose of introducing breaking as a new Olympic event to attract younger generations. In addition, considering the characteristics of the variables set in this study, only those who had watched the Olympics through the media were selected as the sample group. After accounting for potential non-response rates, our survey sample surpasses the specified minimum sample size.

Among the non-probability sampling methods (random sampling method), the judgment sampling method was used to select research subjects who satisfied the above conditions, and face-to-face surveys were conducted to increase the understanding and reliability of the survey contents. Judgmental sampling is a method in which a researcher selects a sample subject that is judged to reflect the opinions of the population well and has the advantage of being representative, which can effectively reflect the characteristics of the population [52]. Before conducting the survey, the researcher first distributed a document explaining the research participation process and a consent form on site and, after receiving written consent, distributed the questionnaire only to participants who agreed to the survey. Next, 520 questionnaires were distributed and collected to directly check whether the respondents had experience watching the Olympics, and the respondents were asked to fill out the questionnaire themselves in a self-reporting manner. Of the collected questionnaires, 493 were selected as the final valid sample after excluding 27 false responses, and the demographic characteristics of the analysis subjects are shown in Table 1. Additionally, this study does not collect or record sensitive information from research participants.

**Table 1:** Demographic characteristic of participants

Variables	Classification	Frequency (N)	Percentage (%)
Gender	Male	290	58.8
	Female	203	41.2
Age	10 s	135	27.4
	20 s	202	41.0
	30 s	156	31.6
Job	Students	214	43.4
	Public official	40	8.1
	Specialized job	60	12.2
	Office job	75	15.2
	Service workers	66	13.4
	Others (housewife, job seekers, unemployed, etc.)	38	7.7
Total		493	100

#### Measurement tool

The measurement tool used in this study was a structured questionnaire that reorganized questionnaire items used in previous studies to meet the purpose of this study. First, for the questionnaire items on newness, the scale used by Lee and Crompton [22], Chang, Wall, and Chu [53], Albaity and Melhem [54], Kim, Kim, and Lee [55], and Lee and Kang [56] was verified for content validity by an expert group and then modified and supplemented to suit the purpose of the study. It comprised seven single-factor questions. The questionnaire items for measuring anticipation comprised four single-factor items by modifying and supplementing the questionnaire items used in Neelamegham and Jain [57], Kim, Jung, and Song [58], and Seo [44]. Media engagement consisted of four single-factor questions by modifying and supplementing the questionnaire items used in Muntinga, Moorman, and Smit [59] and Seo [44]. Finally, the viewing intention for the Olympics comprised four single factors by modifying and supplementing the questionnaire items used in Kim et al. [41] and Choi, Jung, and Lee [8] to suit the purpose of this study. All questions except for participants’ general characteristics (Gender, Age, and Job) were measured using a 5-point Likert scale (1 = not at all, 5 = strongly agree).

### Validity and reliability of measuring

A group of five experts, including two professors of sports management and three PhDs in sports management, verified the validity of the questionnaire [60]. Content validity verification is conducted to confirm the appropriateness and representativeness of the question, to verify whether each question is appropriate for the evaluation of the purpose, and to determine whether the content of the question faithfully represents the content to be measured [52]. To evaluate the content validity of the expert group, the content validity ratio (CVR) proposed by Lawshe [61] and the content validity index (CVI) proposed by Lynn [60] were used. First, the CVR value was calculated by asking a group of experts to respond to the relevance of the items on a 3-point scale (required, usable but not required, unnecessary), and the CVR value = (ne-N/2)/(N/2) (ne = number of experts who responded that it was required, N = total number of experts) was found to be over 0.99. In addition, the CVI value was calculated by asking the expert panel to answer on a 4-point scale regarding how relevant the items were, and the CVI, which is the proportion of experts who answered 3 or 4 for each item, was 0.9 or higher [62], confirming that the content validity was satisfied.

Meanwhile, confirmatory factor analysis (CFA) was performed to assess discriminant validity. CFA verified the discriminant validity of the correlation with other variables, excluding the measurement variable (Table 2)**.** It is, in general, recommended to evaluate CFA using the stable indices of CFI, NFI, TLI, RMR, and RMSEA, which are relatively insensitive to sample size, model fit, and degrees of freedom [52]. In addition, Bagozzi and Dholakia [63] evaluated a good model when the CFI, NFI, and TLI were 0.9 or higher, and the RMR and RMSEA were 0.08 or lower. Based on this, as a result of conducting CFA on the questionnaire items, the goodness of fit of the research model was CFI = 0.970, NFI = 0.959, TLI = 0.956, RMR = 0.053, and RMSEA = 0.074, indicating a relatively excellent model.

**Table 2:** Confirmatory factor analysis

**Variables**	***Questions***	***SC***	***SE***	***t***	**C.R.**	Cronbach’s α'
Novelty	The Olympic introduction of breaking is new	1.000	-	-	0.902	0.909
	The Olympic introduction of breaking breaks the boredom of the traditional Olympics	1.023	0.023	44.479		
	The Olympic introduction of breaking is a departure from stereotypes	0.962	0.026	37.310		
	The Olympic introduction of breaking offers an exciting and surprising experience	0.911	0.024	37.948		
	The Olympic introduction of breaking is intriguing	0.631	0.038	16.400		
	The Olympic introduction of breaking is an opportunity to experience a new Olympic culture	0.316	0.038	8.354		
	The Olympic introduction of breaking has created a new unprecedented perspective on the Olympics	0.474	0.038	12.439		
Anticipation	Looking forward to the 2024 Olympics in Paris	1.000	-	-	0.907	0.923
	Breaking, which was introduced as an official event at the 2024 Paris Olympics, is expected	1.038	0.036	28.823		
	I look forward to watching the breaking competition at the 2024 Paris Olympics	1.161	0.042	27.722		
	We look forward to hearing from you about the 2024 Paris Olympics and breaking events	1.043	0.042	24.302		
Media engagement	Find articles about the 2024 Paris Olympics and breaking	1.000	-	-	0.910	0.926
	Find photos or videos about breaking, which was introduced as an official event at the 2024 Paris Olympics	0.928	0.036	25.840		
	Find information about the 2024 Paris Olympics and breaking	0.972	0.037	26.130		
	Find social media related to the 2024 Paris Olympics and breaking	1.032	0.036	28.281		
Viewing intentions	During the Paris Olympics in 2024, the media will watch the game	1.000	-	-	0.866	0.884
	During the 2024 Paris Olympics, news related to breaking will be confirmed through the media	0.978	0.038	25.883		
	During the 2024 Paris Olympics, we will look for related websites or social media	0.951	0.039	24.527		
	I will try to watch the breaking competition during the 2024 Paris Olympics	0.797	0.044	18.147		

*χ*^*2*^ = 439.395 (*df* = 118,* p* = 0.000), CFI = 0.970, NFI = 0.959, TLI = 0.956, RMR = 0.053, RMSEA = 0.074

#### Data analysis process

The questionnaire used for the final analysis was the result of data analysis using Windows PC/SPSS 28.0 and AMOS 25.0 after coding and error reviews. First, the general characteristics of the study participants were analyzed using frequency analysis. Second, CFA was performed to verify all factors, and reliability was verified by calculating Cronbach’s α coefficient to ensure internal consistency. Finally, a correlation analysis was performed to analyze the relationships between the variables, and structural equation modeling was performed to derive a structural model.

## Results

### Correlation analysis

A correlation analysis was performed to confirm the correlations between the variables. The results of the correlation analysis between each variable demonstrated no multicollinearity problem because no variable showed a correlation of 0.8 or higher in the range of the correlation coefficient values of 0.693 to 0.767 (Table 3).

**Table 3:** Correlation analysis

Variables	1	2	3	4
Novelty^1^	**0.623**			
Anticipation^2^	0.752^**^ (0.566)	**0.710**		
Media engagement^3^	0.693^**^ (0.480)	0.767^**^ (0.588)	**0.715**	
Viewing intentions^4^	0.700^**^ (0.490)	0.712^**^ (0.507)	0.741^**^ (0.549)	**0.621**

^**^p < 0.01, () is the square value of the correlation coefficient, and the shaded part is the AVE

Fornel and Larcker [64] suggested that discriminant validity could be secured if the AVE value was larger than the squared value of the correlation coefficient; the largest square value of the correlation coefficient was 0.767 (= 0.588), and the smallest value of the AVE was 0.621, ensuring discriminant validity.

#### Model verification

The results of the analysis verified the suitability of the structural model established in this research: *χ*^*2*^ = 416.923 (*df* = 117, *p* = 0.000), CFI = 0.972, NFI = 0.961, TLI = 0.958, RMR = 0.038, and RMSEA = 0.072. According to Kline [65], when the indicators of CFI, NFI, and TLI, which generally evaluate the overall fit of a model, are above 0.8 to 0.9, the RMR and RMSEA are evaluated as good when they are less than 0.08. Therefore, it was confirmed that this research model explained the research hypothesis and that the empirical dataset was suitable for adoption (Table 4).

**Table 4:** Fit index of research model

**A construct**	***χ***^***2***^	***df***	***p***	**CFI**	**NFI**	**TLI**	**RMR**	**RMSEA**
Acceptance level	416.923	117	.000	0.972	0.961	0.958	0.038	0.072
Acceptance criteria	-	-	-	more than 0.8 ~ 0.9	more than 0.8 ~ 0.9	more than 0.8 ~ 0.9	less than 0.08	less than 0.08

#### Hypothesis testing

A structural equation model was analyzed to confirm the causal relationship between the research hypotheses and the variables in the research model (Table 5). The results of the hypothesis testing analysis indicated the following: First, the path coefficient of H1 was 0.807 (t. Second, the path coefficient for H2 is 0.826 (*t* = 18.144, *p* < 0.001), which was accepted. Third, the path coefficient for H3 is 0.146 (*t* = 8.748, p 0.001), which was accepted. Fourth, the path coefficient of H4 was 0.160, which was rejected. Fifth, the path coefficient for H5 is 0.885 (*t* = 8.791, *p* < 0.001), which was accepted. The path coefficient of H6 is 0.184 (*t* = 2.094, *p* < 0.01), which was accepted.

**Table 5:** Hypothesis testing results

H		Path	*SE*	*CR*	*p*	Accept/Reject
H1	Novelty → Anticipation	0.807	0.199	8.748	0.000^***^	accept
H2	Novelty → Media engagement	0.826	0.053	18.144	0.000^***^	accept
H3	Novelty → Viewing intention	0.146	0.100	3.674	0.001^**^	accept
H4	Anticipation → Viewing intention	0.160	0.097	1.816	0.125	reject
H5	Anticipation → Media engagement	0.885	0.094	8.791	0.000^***^	accept
H6	Media engagement → Viewing intention	0.184	0.088	2.094	0.001^**^	accept

^**^p < 0.01

^***^p < 0.001

## Discussion

### Summary of results

This study empirically analyzes the relationship between novelty, anticipation, media engagement, and viewing intention, focusing on the change in the perception of potential viewers about breaking, which will be introduced as a new event at the 2024 Paris Olympics. Based on the results, the following conclusions are drawn.

First, the novelty of introducing breaking as an Olympic event had a statistically significant effect on the anticipation of potential Olympic viewers. In this regard, McIntosh et al. [24] identified the mutual influence relationship between novelty and anticipation, and Nunnally and Leonard [66] revealed that a strong desire for a new object, situation, or environment led to high anticipation of what could satisfy it. In addition, Han and Lee [67] argue that as consumers' desire for novelty increases, their anticipations for specific consumption behaviors also increase, and Choi, Jeong, and Lee [8] show results consistent with this study, stating that novelty created by introducing a new Olympic sport increases anticipations for the Olympics. As the IOC recently recognized subcultures such as 3:3 basketball, BMX freestyle, skateboarding, sports climbing, and surfing as mainstream cultures, as well as breaking, these results are considered to have a synergistic effect that raises young generations’ anticipations for the Olympics. Therefore, it is necessary to establish an effective promotional marketing strategy that can strongly stimulate the novelty of breaking to potential Olympic viewers and raise their anticipations. According to Kim et al. [68] most of the new sports introduced in the 2020 Tokyo Olympics did not have famous athletes, and the sports themselves were unfamiliar as sports; consequently, they are pushed down in the priority and order of domestic broadcasting, making it difficult to guarantee high viewership ratings. As most of the new Olympic sports are largely unknown to the public and can only be viewed online [7], the IOC must first find various promotional methods to expose new Olympic sports to the Millennials and Gen Z as much as possible.

Second, the novelty of introducing breaking at the Olympics has a statistically significant effect on the media engagement of Olympic viewers. These results were consistent with those of Kim and Kim [69], who revealed the relationship between media participation and novelty, Jeong [70], who mentioned the novelty of Olympic media participation, and Jeong and Kim [71], who reported that new experiences acted as a direct antecedent of participation and provided theoretical support for this study. In other words, the younger generation perceived the novelty of introducing breaking as a new event as a novel experience that shatters stereotypes about the Olympics, suggesting that this can lead to an increase in media engagement.

Third, the novelty of introducing breaking as an Olympic game had a statistically significant effect on the viewing intentions of potential Olympic viewers. Accordingly, Park and Park [72], Lepp and Gibson [73], and Assaker, Vinzi, and O'Connor [74] reveal that new experiences play a pivotal role in the consumption behavior step, while Kim, Kim, and Lee [55] and Jong [70] reveal that the desire for novelty is a major variable in consumer decision-making in sports situations. Taken together, this suggests that the novelty of breaking, introduced as an official event for the 2024 Paris Olympics, is a strong factor in inducing anticipation, media participation, and viewing intentions for the Olympics among potential viewers of the younger generation. For example, e-sports have proven their popularity beyond traditional sports by being adopted as an official event in the 2022 Hangzhou Asian Games. E-sports, which was formed by adding sports elements to games, a representative play culture of the Millennials and Gen Z, has grown through broadcasting that allows fans to become enthusiastic about the gameplay of professional players and form fandoms through various OTT platforms even if they do not directly participate in the game [75, 76]. Likewise, if breaking makes good use of its unique elements such as advancement in IT technology, Millennials and Gen Z friendliness, globality, and a harmony of sports and entertainment elements, it can become a sustainable sport.

Fourth, anticipation of the introduction of breaking did not have a statistically significant effect on the viewing intentions of potential Olympic viewers. These results by Lee and La [77], Chen [78], and Hsu, Chang, and Chen [42], who state that consumer anticipation has a priori and predictive character and serves as evaluation criteria for feeling satisfied or dissatisfied, including those by Ahn [43] and Seo [44], which identify a statistically significant relationship between anticipation for mega sporting events and sports participation behavior, are contrary to the findings of this study. In the case of South Korea, where this study was conducted, it was judged that exerting influence in the process of explaining the relationship between expectations of the Olympics and viewing intentions was difficult because of a lack of information and publicity for new Olympic events, such as breaking.

Fifth, anticipation of the introduction of breaking as an Olympic event has a statistically significant effect on the media engagement of potential Olympic viewers. Accordingly, Brakus, Schmitt, and Zarantello [79] state that media engagement has a positive effect on consumer experience, value, anticipation, and attitude. Lee and Chang [80] found that the higher the media engagement, the greater the anticipation of social media. In addition, previous studies have stated that strong expectations appear as strong engagement, and the results of this study have the same theoretical context [46]. These findings suggest that, to increase the level of media engagement of the younger generation—potential viewers of the Olympics—, their anticipation should be formed first. For example, considering the characteristics of the younger generation, who are familiar with the media, if we increase the frequency of exposure to various information (host country, stadium, schedule, game rules, player information, etc.) related to new Olympic events, such as breaking and centering on the media, it will be effective in forming their anticipation.

Finally, media engagement had a statistically significant effect on the viewing intentions of potential Olympic viewers. Kim et al. [50] and Kim and Son [51] reveal that media engagement has a direct effect on usage behavior, and Lim et al. [49] have proved an effective relationship between media engagement and Olympic viewers sports channel viewing behavior. In addition, Higgins [81] supports the results of this study by suggesting that engagement is an effective factor in determining liking or experiencing a specific situation. Consequently, it is reasonable to assume that inducing a high level of media engagement among the younger generation, potential viewers of the Olympics, affects actual viewing intention. To this end, it is necessary to focus on eliciting smooth interactions between the Olympics and viewers by inducing sharing, chatting, and commenting on media content related to the former and promoting interactive communication activities with the latter.

### Limitations and future studies

This study has several limitations. First, as this study limited the research subjects to the younger generation aged 17–30 years living in Seoul and Gyeonggi-do among 17 cities and provinces in Korea, expanding and interpreting the results of this study to all potential viewers of the Olympics will result in a generalization error. Therefore, it is necessary to conduct in-depth research that reflects the characteristics of various age groups by expanding the scope of the research subjects. Second, this study focused on clarifying the relationship between novelty, expectation, media engagement, and viewing intention for the Olympics only for breaking among the new events introduced at the 2024 Paris Olympics; differences existing because of the specificity of other new sports events were not considered. Therefore, in follow-up studies, it is necessary to expand the range of new sports and design a comprehensive study that considers the specificity of each sport.

Finally, in addition to the variables set in this study, there were limitations in identifying various interactions that could affect the viewing intentions of potential Olympics viewers. Therefore, an integrated study using research methods, such as participatory observation and in-depth interviews, that can provide an in-depth understanding of the various opinions that can affect the viewing intentions of younger generations and potential viewers of the Olympics is required.

## Conclusion

In summary, the younger generation perceived breaking, which was introduced as a new sport at the 2024 Paris Olympics, as a new, novel, and fresh sport that was different from the traditional Olympic image. This result is in line with what the IOC intended as human nature reacts more sensitively to new stimuli than to familiar ones. Based on the results of this study, it is suggested that a change in perception according to the rapidly changing characteristics of the young generation and consumption trends must be premised to attract potential viewers to the Olympics.

With the 2024 Paris Olympics about two years away, the values that the younger generation wants to experience through the Olympics are not traditional collectivistic values based on nationalism, but they feel catharsis through the courage and challenge of athletes participating in the Olympics, which has changed its value as a festival enjoyed with costumes and exciting music. Therefore, the IOC should pursue an internal change strategy that provides and emphasizes the Olympic values they seek by prioritizing a deep understanding of the characteristics and rapidly changing consumption patterns of the younger generation as well as simple external changes, such as the introduction of new Olympic events.

## Supplementary Information

Supplementary Material 1.

## Data Availability

All data generated or analyzed during this study are included in this published article [and its supplementary information files].

## Abbreviations

IOC: International Olympic Committee
KPSA: Korea Professional Sports Association
CVR: Content Validity Ratio
CVI: Content Validity Index
CFA: Confirmatory Factor Analysis
